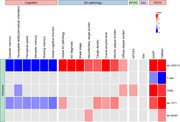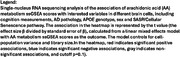# Single cell nuclei transcriptomics of human brains identify a role for homeostatic microglia in cellular senescence in Alzheimer’s disease

**DOI:** 10.1002/alz.087171

**Published:** 2025-01-03

**Authors:** Hussein N Yassine, Boyang Li, Shaowei Wang, Brandon Ebright, Marlon Vincent V. Duro, Bilal Ersen Kerman, Isaac Asante, Zoe Arvanitakis, Stan G Louie

**Affiliations:** ^1^ USC Keck School of Medicine, Los Angeles, CA USA; ^2^ University of Southern California, Los Angeles, CA USA; ^3^ USC School of Pharmacy, Los Angeles, CA USA; ^4^ Rush Medical College, Chicago, IL USA

## Abstract

**Background:**

Cellular senescence is a hallmark of aging and has been implicated in several neurodegenerative diseases including Alzheimer’s disease (AD). Senescence cells undergo changes in gene expression and metabolism and can exhibit a so‐called “senescence‐associated secretory phenotype” (SASP) characterized by increased secretion of pro‐inflammatory molecules and factors which can damage nearby cells, contributing to AD pathology progression.

**Method:**

In this study, we determined mechanisms of cellular senescence using human postmortem brain samples, cellular models, and APOE4 animal models. Bulk (n = 632) and single‐cell nuclei transcriptomic profiling (n = 427) of the human dorsolateral prefrontal cortex (DLPFC) from the Religious Order Study/Memory Aging Project (ROSMAP). Lipidomic profiling was performed on a subset of 200 brains from the midfrontal cortex of ROS.

**Result:**

Our findings revealed upregulation of cellular senescence signatures in postmortem AD brain tissues across different cell types in comparison with controls. We identified a strong correlation between SASP and arachidonic acid (AA) metabolism (P<0.001) in bulk RNA. In single cell nuclei transcriptomics, AA Activation was strongly correlated with P2RY12 (homeostatic) microglia (P<0.0001), and was associated with worse performance on all cognitive domains (p<0.001) and AD neuropathology (P<0.001) as shown in the figure. Lipidomic analysis of postmortem brain tissues confirmed activation of AA derived eicosanoids. Pathway analysis implicated the activation of calcium dependent phospholipase A2 (cPLA2). Inhibiting cPLA2 by treatment with ASB14780 reduced senescence‐associated eicosanoids in APOE4 mouse models.

**Conclusion:**

This work implicates the sustained activation of homeostatic microglia as an underlying mechanism of cellular senescence in the AD brain.